# Infection-Related Focal Segmental Glomerulosclerosis in Children

**DOI:** 10.1155/2016/7351964

**Published:** 2016-05-17

**Authors:** Anne Katrin Dettmar, Jun Oh

**Affiliations:** Department of Pediatric Nephrology, University Children's Medical Clinic, University Medical Center Hamburg-Eppendorf, 20246 Hamburg, Germany

## Abstract

Focal segmental glomerulosclerosis (FSGS) is the most common cause of steroid resistant nephrotic syndrome in children. It describes a unique histological picture of glomerular damage resulting from several causes. In the majority of patients the causing agent is still unknown, but in some cases viral association is evident. In adults, the most established FSGS causing virus is the human immune-deficiency virus, which is related to a collapsing variant of FSGS. Nevertheless, other viruses are also suspected for causing a collapsing or noncollapsing variant, for example, hepatitis B virus, parvovirus B19, and* Cytomegalovirus*. Although the systemic infection mechanism is different for these viruses, there are similarities in the pathomechanism for the induction of FSGS. As the podocyte is the key structure in the pathogenesis of FSGS, a direct infection of these cells or immediate damage through the virus or viral components has to be considered. Although viral infections are a very rare cause for FSGS in children, the treating pediatric nephrologist has to be aware of a possible underlying infection, as this has a relevant impact on therapy and prognosis.

## 1. Introduction

Focal segmental glomerulosclerosis (FSGS) describes a typical histologic pattern resulting from different glomerular diseases ending in almost the same morphological picture. Children with FSGS usually present with severe nephrotic syndrome. However the main cause for childhood nephrotic syndrome is minimal change disease, which usually responds to treatment with steroids in 70–80%, but FSGS is the most frequent glomerulopathy seen in children with steroid resistant nephrotic syndrome. Although there might be some common factors underlying the pathogenesis of the classical histological pattern [1] independent of the causing agent, there are also some mechanisms, which differ in the initiation of FSGS, regarding the trigger.

Concerning the cause of FSGS and, often more importantly, the therapeutic approach, primary idiopathic and secondary forms have to be distinguished clearly. The term primary FSGS is reserved for forms of FSGS with primary podocyte injury, where no cause for the histopathologic lesions can be found. There is evidence that a circulating factor might be responsible for the development of the disease [2, 3]. In secondary FSGS there is a typical histological segmental pattern, which occurs in reaction of several different causes.  Table 1 summarizes the important causes of secondary FSGS. Furthermore secondary FSGS includes genetic mutations in podocyte proteins, which are common reasons for steroid resistance in nephrotic syndrome [3]. The different causes are important to be distinguished and have to be carefully examined, as there are mostly different therapeutic strategies. The course of secondary FSGS is often more chronic, compared to the usually fast progression and immediate onset of primary FSGS [1]. Independent of the underlying cause of many forms of FSGS, it was shown in the last years that there is a genetic susceptibility for the development of this kidney disease [4].

Besides others, viral infection seems to be a prominent trigger for the development of the classical histological form of FSGS. Although secondary FSGS due to viral infections is not that common in children, it is very important that they have to be carefully excluded before the initiation of any treatment.

## 2. Pathomechanism of Virus-Related FSGS

The most common forms of virus-related FSGS are associated with HBV, HCV, and HIV in adults. Infections with CMV and parvovirus B19 seem also to play a role, but the causality is sometimes hard to prove [5]. The criteria to link the acute onset of FSGS to acute viral infections are very complex and difficult. Serological diagnostics, detection of specific antigens in the blood or in any other body specimen, and the identification of viral antigens in glomerular structures together with the clinical and histological picture contribute to the diagnosis of virus-related nephropathy. The pathogenic triggers between a viral infection and the initiation of FSGS also seem to be evident, when the renal disease improves after eradication of the virus [6].

The pathomechanism of viral nephropathy depends on the type of the virus and the glomerular disease. In the acute forms of infection-associated FSGS the initiation of the nephropathy seems to be the direct effect of the infection of glomerular cells [6, 7], while the more chronic forms of virus-related nephropathies seem to be linked to the formation and deposits of various immune-complexes, which are formed* in situ* or circulate throughout the body [6, 8]. In addition, various viral proteins can often induce the synthesis of mediators that also induce sclerosis and may worsen the ongoing nephropathy [9, 10].

In FSGS, the podocyte is standing in the center of the development of the disease. In virus-related FSGS the direct infection of podocytes might be one of the key mechanisms. Hints to support this hypothesis have been published for HIV-associated nephropathy (HIVAN) [11], showing that the expression of HIV genes in podocytes alone led to the development of HIVAN.

Besides the classical histological form of FSGS, there are other variants, such as the so-called collapsing variant of FSGS. This variant most notably is associated with HIV infection but there are also forms of HIV-negative “idiopathic” collapsing FSGS. In the literature, it has been associated with several conditions, including HIV infections, as well as with mycobacterium tuberculosis, filariasis, leishmaniasis, and campylobacter. For most of the associations the numbers of cases are still few, and pathogenesis is not yet fully understood. Thus it is not clear whether these infections are coexisting conditions or cause of the development of FSGS. The specific histological pattern of FSGS accounts only for a small proportion of virus-related glomerulopathies.

## 3. Virus-Related FSGS in Children

Minimal change nephrotic syndrome can be associated with several viral infections [12]. Even though symptomatic viral and bacterial infections are much more common in children compared to adults, virus-associated FSGS is still rare. Consequentially available pediatric data are limited. While some studies have described the appearance of membranous glomerulopathies due to hepatitis B virus infection in children, patient's cases of FSGS due to any viral infection have rarely been reported. Nevertheless, it is important to exclude a viral infection prior to the treatment of any nephropathy, as the standard therapy is often an immunosuppressive therapy. However, the positive proof of any microbes should still be considered with caution, because a direct link for most of the viruses mentioned below has not been proven yet. Therefore, it is important to consider screening for genetic mutations as the cause for the development of FSGS.

## 4. HIV-Related FSGS

Seen from the epidemiologic perspective, the human immunodeficiency virus (HIV) is one of the most important infections in the world, with prevalence from 0.2 percent in Western Europe to 5 percent in Sub-Saharan Africa in 2010 [13]. By the end of 2011, 3.4 million children were infected worldwide with the HIV. In particular, in Africa, perinatal transmission from untreated HIV-positive women to their children is a large source of new infections, even though the infection rate was reduced after the introduction of antiretroviral therapy. Before this therapy was available, children had a mortality rate over 50% within the first two years of their life [14]. Nowadays, the long-term survival is much better, but it leads to the need of treatment of comorbidities such as severe kidney involvements. There are four groups of glomerulopathies occurring in the course of HIV infection: HIV can cause classical HIVAN, which shows histological hallmarks of FSGS with collapse of the glomerular tuft or mesangial hyperplasia. Besides the classical form HIV also can induce diffuse proliferative or lupus-like glomerulonephritis with mesangial immune deposits, thrombotic microangiopathy, or a more heterogeneous group which includes immunotactoid glomerulonephritis [15]. Based on a cohort-study in the United States, it has been estimated that the incidence rate for HIV-associated nephropathy is about 2.6 per 100 patient-years [16, 17]. The typical collapsing form of FSGS associated with the HIV infection is more common in patients of African ethnicity. It is the most common cause of kidney disease in HIV-infected children and adolescents in countries such as South Africa and Nigeria [18, 19]. The reason for this high rate of HIVAN is the common mutation in the APOL1 gene, which is required for the development of particularly this form of FSGS [20]. There are other forms of HIV glomerulopathies, which are more common in different regions of the world [21].

The histological picture of HIVAN is the collapsing variant of FSGS and regularly has coexistent affection of glomeruli and tubuli. It usually shows hyperplasia or hypertrophy of podocytes, sometimes with detection of protein inclusions in the cytoplasma [22]. The term “collapsing” refers to the retraction of the basement membrane with resulting collapse of the capillaries with widening of Bowman's space. The whole glomerulum or only segments are affected by sclerosis, and some of them might even show degenerative changes or necrotic areas [23, 24]. Microcystic dilation, interstitial fibrosis, tubular cell proliferation, and apoptosis occur in the tubulointerstitium [25]. However, a noncollapsing form of FSGS with or without mesangial hyperplasia in combination with microcystic tubular dilation and interstitial inflammation occurs more frequently in children [21]. Besides many efforts the pathogenic role of the virus itself in HIVAN is still not totally clear, but it is currently hypothesized that the changes are caused by the infection of renal cells by HIV or due to a direct toxic effect of specific viral proteins [22]. Experimental studies have shown that the virus has immediate toxic influence on renal cells* in vitro* [9]. HIV-RNA has been detected in renal biopsies in patients with HIVAN [26]. In HIV-1 transgenic mouse models a direct link between the expression of HIV-1 in the kidney and the development of HIVAN was documented [22]. Zhong and colleagues showed an immediate effect of the HIV and viral components targeting podocytes. The study indicates that the expression of HIV gene products alone is already sufficient to develop HIVAN [11]. Besides the direct cytopathic effect of virus or viral components, there might be some other indirect effects on the kidney. For example, the uptake of circulating virally encoded molecules in kidney cells and the immediate release of cytokines by infected lymphocytes or monocytes may harm kidney cells [22].

Since the last few years, there is a growing body of evidence that a mutation in APOL1 gene is required for the development of HIVAN. The APOL1 G1 and G2 genetic variant can only be detected in patients from African descent [20]. Through different ways of trypanolytic activity the G1 or G2 variant prevents acute trypanosomiasis in humans [27]. The exact mechanism of this mutation leading to a higher susceptibility for HIVAN and other forms of FSGS [28] is not definitely identified yet. APOL1 is one of the 6 members of APOL gene family, located on chromosome 22. APOL1 is secreted from various cells and circulates in the blood. Nevertheless it still remains unclear whether the APOL1 which is circulating or the APOL1 produced and expressed in the kidney is responsible for the kidney damage [29]. On one hand, the uptake of APOL1 G1 or G2 risk isoforms seems to contribute to podocyte death* in vitro* and therefore a direct toxic effect of the mutated isoforms is discussed [20, 30, 31]. On the other hand, there are studies in renal allografts, showing that the kidney expressed local APOL1 is the reason for kidney damage and not the circulating form of APOL1: the APOL1 mutation in transplant recipients did not impair the graft survival, when kidneys of non-APOL1 mutants were transplanted [32]; however kidneys from donors with two risk alleles had a shorter survival time when they were transplanted into patients with no APOL1 risk mutation [33]. In the glomeruli, APOL1 seems to play an important role in autophagy and autophagic homeostasis in podocytes. In patients, homozygous for a G1 or G2 mutation, HIV (or another additional trigger) might serve as a second hit [34]. It has been theoretically postulated that the mutated isoforms of APOL1 cannot be inactivated by another unknown factor, but this has not yet been proven yet. Mutated APOL1 can lead to apoptosis, autophagy, and cell death, probably because of a mechanism related to trypanosome killing (e.g., destabilization of lysosomal membrane) [29]. Additionally Lan and colleagues showed that there is a secretion of mutated APOL1 from smooth muscle cells in HIV milieu, which also could contribute to direct podocyte damage [35].

Besides the important mutation in APOL1, there seem to be a lot of other genetic factors, predisposing for the development of HIVAN in children [34], and recently APOL1 mutations were also discussed to induce preeclampsia in a transgenic mouse model [36]. There is an ongoing discussion about the precise role of productive mesangial cell infection by HIV-1 [37], especially in children. In some cases, children with HIV-associated proteinuria initially present with “only” mesangial hyperplasia, which shows slower progression, compared to classical HIVAN [27]. Besides the viral effects, influence of antiretroviral therapy, antibiotics, antifungals, anti-inflammatory drugs, and the combination of various drugs may also contribute to different forms of kidney disease in HIV-infected children [21].

Patients with HIVAN usually present with nephrotic range proteinuria and renal insufficiency. In ultrasound, they show enlarged, highly echogenic kidneys [6]. Compared to sometimes very fast progression in adults, initially there is slow onset as well as milder clinical course of the noncollapsing form of FSGS in children. Overall there is slower progression to end-stage renal disease in the pediatric population [37, 38]. Therefore, screening for (micro)hematuria and proteinuria in HIV-positive children is important for the timely discovered onset of glomerulopathies. As biopsies are not always performed in HIV-positive children, a combination of persistent proteinuria, microcysts in urine sediment (shed epithelial cells), and highly echogenic kidneys can be used for the clinical diagnosis of HIVAN in children [21]. The renal progression can be delayed by an intensive and effective retroviral therapy. When there is proof of kidney involvement in HIV-positive patients, then there is an indication to start or intensify the highly active antiretroviral therapy (HAART) based therapy [39, 40]. This might be helpful to stop the progression of the renal disease, although there are several well-described significant nephrotoxic side effects of the therapy [21]. The introduction of HAART, which was introduced in 1996, has dramatically reduced the incidence of HIVAN [41, 42]. Even though the therapy of HIV improved over the decades, the renal outcome in HIVAN is still poor compared to other HIV-associated renal lesions. The mean time in children from the initial detection of proteinuria to end-stage renal disease (ESRD) varied between 8 months and 3 years without HAART. Nearly half of the patients, diagnosed with HIVAN, developed ESRD within two years. Nevertheless, the use of antiretroviral therapy delayed the initiation of renal replacement therapy [21, 41]. Overall the survival of children is better compared to adults with HIVAN [43]. Therefore, it is very important to survey kidney involvement of HIV children and to treat HIV-infected patients according to the actual guidelines [44].

## 5. Hepatitis B Virus-Related FSGS

Worldwide, there are approximately 240 million people infected with the hepatitis B virus (HBV) [45]. This infection is associated with a diverse range of hepatic damage. Most chronic infected patients acquire their infection around the time of birth or in the first years of life [46]. It has been reported that chronic HBV infections are associated with several types of glomerulopathies. It is most common in children with minimal change disease [47], membranous nephropathy, and IgA nephropathy [6]. But there are also some reports referring to HBV-associated FSGS in adults [6, 48, 49]. HBV is a hepatotropic double-stranded DNA virus, which is* per se *not cytopathic. The hepatitis develops because of the immune-mediated reaction. Still, little is known about the pathogenesis of FSGS and other nephropathies associated with a chronic HBV infection. Even though there are significant differences between HIVAN and HBV-associated nephropathy, there is growing evidence for a related pathophysiological mechanism [48]. Sakai and colleagues detected HBV-DNA in urinary podocytes in a patient with chronic HBV infection and collapsing variant of FSGS, indicating podocytes as a possible direct target. The expression of HBV-DNA was significantly reduced after the effective treatment with entecavir, which was associated with a histological recovery of the FSGS pattern and an improvement of proteinuria [48]. The diagnosis of HBV-associated FSGS includes the serologic presence of HBV antigens or antibodies, the detection of at least one or more HBV-related antigens in immune-histochemistry, and the absence of other causative reasons [6, 21]. In children forms of HBV-associated FSGS are still rare. Most of the affected children show a mild membranous nephropathy, membranoproliferative glomerulonephritis, or IgA nephropathy and present with a nephrotic or nephritic syndrome [50].

Furthermore, the treatment of HBV-related nephropathy remains controversial. Children seem to have a high rate of spontaneous remission [50]. The use of steroids, which are used in the idiopathic forms of the disease, may induce a higher replication rate of HBV [51, 52]. Even though concurrent, there are some reports for a benefit of steroids in membranoproliferative glomerulonephritis or membranous nephropathy [6]. For HBV-associated FSGS the eradication of the virus seems to be critical for the recovery of the FSGS lesions [48]. The lamivudine therapy in HBV-associated membranous nephropathies led to a complete remission of nephrotic syndrome in some children and adults [53]. There is only limited data about the treatment of FSGS due to an HBV infection, especially for children. But in reported cases the treatments with lamivudine [49, 54] or entecavir [48] were effective in the reduction of viral load and were leading to an improvement of the kidney disease. Compared to other infection-associated glomerulopathies, the prognosis in hepatitis B induced renal lesions is over all quite good [50].

## 6. Hepatitis C Virus-Related FSGS

The hepatitis C virus (HCV) is a small RNA virus. Its replication is confined to the liver, but there are a variety of extra hepatic disease manifestations, including mesangiocapillary glomerulonephritis type I [6]. But there are also some single-case reports, describing different glomerulopathies, including FSGS.* De novo* glomerulopathies in transplanted kidneys are often associated with HCV infections. However, HCV-associated renal manifestations are pretty rare in children [6]. Because HCV-related FSGS is so infrequent and HCV glomerulopathies occur only in adults, this review will not focus on this form of FSGS.

## 7. Parvovirus B19-Related FSGS

The seroprevalence of parvovirus B19 (PVB19) reaches up to 80 percent in older age groups [55]. The infection with PVB19 is associated with a wide range of clinical symptoms, and the severity of the disease depends on the hematological and immunological status of the infected individual. Although an infection in healthy children only induces the benign erythema infectiosum or remains asymptomatic, in immune-incompetent children (or adults) and patients with, for example, sickle cell disease, PVB19 can lead to severe aplastic anemia and glomerulonephritis [56, 57]. Furthermore, an acute infection of a pregnant woman can lead to vertical transmission, which then can cause intrauterine death, hydrops fetalis, or neurological manifestations [58, 59].

PVB19-associated glomerulonephritis usually occurs in the second or third decade and shows the histological pattern of mesangiocapillary glomerulonephritis or endocapillary glomerulonephritis. But there is also a collapsing variant of FSGS, which can be associated with PVB19 [6, 60]. This collapsing FSGS, which also is predominantly seen among people of African American origin, is similar to the lesions in HIVAN, but these patients are HIV-negative. This variant is also described as the idiopathic form of collapsing FSGS. PVB19-associated FSGS in children is very rare but there are some few case reports showing that an infection with PVB19 in children with homozygous sickle cells disease or healthy adolescent can lead to FSGS [56, 61]. Besides the idiopathic collapsing FSGS PVB19 can also be associated with* de novo* collapsing FSGS in transplant recipients [62, 63].

Although there is no evidence that the infection itself can cause glomerulonephritis, the increased prevalence of PVB19 DNA in patients suffering from idiopathic or collapsing FSGS indicates a pathogenic role for PVB19 in the development of renal disease [6, 64] even though the causality has not been proven so far.

In collapsing FSGS the prevalence of virus DNA in blood and in biopsies is higher compared to controls [6, 65] and it also seems to be higher in the collapsing variant compared to noncollapsing FSGS forms [60]. It has been already speculated that some patients, unable to generate an effective immune response against viral infections, have higher rates of viremia, which again increases the risk for the development of PVB19-associated diseases. In immune-incompetent patients PVB19 may persist and induce variants of kidney disease [57]. The histological picture is similar to that in HIVAN. There are characteristic glomerular changes, including increased size and number of visceral epithelial cells, cytoplasmic vacuoles, and protein absorption droplets [66]. Moudgil and colleagues also have demonstrated PVB19 DNA in podocytes and in tubular and parietal cells. The affected podocytes were morphologically impaired [60]. This leads to the hypothesis that, similar to the pathogenesis in HIVAN, PVB19-related collapsing FSGS can also affect the podocytes directly. PVB19 binds to the GB4 in erythrocytes [67], which is also expressed in the kidney [68] and in cultured human podocytes [69].

Patients with idiopathic collapsing FSGS usually present with hypertension, proteinuria, nephrotic or nephritic syndrome, and elevated levels of creatinine [70]. There is no evidence-based therapy for collapsing FSGS in HIV-negative patients. The current therapies are empiric and include strategies from therapy of the noncollapsing idiopathic form. The therapeutic regime includes steroids, cyclophosphamide, and cyclosporine [71]. Additionally, there is one report of a successful treatment with rituximab [72] in a cyclosporine resistant patient. Nevertheless, the achieved remission rate is not satisfying yet and according to the literature not above 10% [70, 71]. In particular steroids are not useful among patients of African American origin [73, 74]. The prognosis of* de novo* FSGS after transplantation, in which infection with PVB19 is a risk factor, is still poor [63]. While the majority of these patients will develop ESRD after transplantation, the role of the virus infection is still not fully understood.

Data regarding therapy and prognosis especially for children are limited. One patient out of 5 pediatric patients with homozygous sickle cell disease and glomerulopathy developed ESRD, and another patient received a complete remission [56].

## 8. Cytomegalovirus-Related FSGS

Cytomegalovirus (CMV) is highly prevalent and is reaching seropositivity in up to 90 percent of the healthy population [75, 76]. In immunocompetent patients CMV infection is usually completely discreet, but it plays an important role in the development of congenic malformations in pregnant women with a CMV seroconversion [77]. CMV is the most common congenital infection in human. The majority of the infections in newborns are asymptomatic, but 10% of the children develop cytomegalic inclusion diseases with up to 30% mortality. Kidney involvement in congenital or perinatal CMV infection is rare and the causality is not proven yet [78, 79].

In later acquired CMV infection FSGS is pretty uncommon, but there are some primary cases published [80]. Once a patient is infected, CMV can never be totally eliminated [81]. The pathogenesis of CMV-related glomerulopathies, especially FSGS, has not been sufficiently understood yet. A podocyte specific infection, as it occurs in HIVAN or in HBV, has not been shown so far. It is suspected that the podocyte damage occurs because of the specific T-cell reaction against the virus [81]. Glomerulopathies associated with an acute CMV infection are seen together with distinctive intracellular structures, so-called tubuloreticular inclusions, within lymphocytes and endothelial cells. These inclusions are specific markers of a systemic stimulation by cytokine interferon. The histological pattern varies from tip version to collapsing variant of FSGS [80, 82, 83]. Even though the direct proof of CMV-DNA was not always possible (or done) in these cases, there are strong indications that an acute CMV infection can induce FSGS. All cases with a CMV-related FSGS were found in adults, with the exception of only one 16-year-old girl [82]. In the cited case reports, therapy was mainly based on glucocorticosteroids and an antiviral therapy, which seemed to be beneficial and lead to remission in the cited patients.

## 9. Epstein-Barr Virus-Related FSGS

Similar to CMV and PVB19, Epstein-Barr virus (EBV) has high seropositivity (up to 98 percent) among healthy western population. In many adult and adolescent patients seroconversions stay unnoticed. In teenagers it can cause infectious mononucleosis (also referred to as “kissing disease”). But EBV infection has also been associated with several renal diseases in 3–16 percent of all cases of infectious mononucleosis [84, 85]. Further EBV can cause interstitial nephritis [86, 87], IgA nephropathy [88], crescentic glomerulonephritis [89], and membranous nephropathy in children [90]. There is only one report about collapsing FSGS association with an acute EBV infection in a young woman [91].

The mechanism of glomerular damage after EBV infection is not yet elucidated. But it was assumed by Joshi and colleagues that there might be an uptake of immunoglobulin-EBV complexes by the glomerular cells, similar to the cells in the nasopharyngeal mucosa. In this case splenectomy was performed because of unremitting abdominal pain. Within a few days after the surgery the renal dysfunction resolved. After the acute phase of the disease proteinuria improved but did not reach normal values. However, EBV-DNA was not detected in the kidney in this particular patient [91]. Therefore the causality of EBV in the development of FSGS has still to be proven.

## 10. Summary and Conclusion

There are some viral infections, which are most likely causing glomerulopathy, but the causality has not been proven for all of them. Even though virus-related FSGS is not very common in children, pediatric nephrologists should always consider the possibility that the impairment of renal function in a patient may be caused by an infection. Therefore, a carefully taken medical history, a correct physical examination, and a well-conceived diagnostic approach will help to distinguish between primary and secondary FSGS and help to choose the appropriate therapeutic strategy. The renal prognosis of FSGS related to infections varies and is dependent on the underlying type of infection.

## Figures and Tables

**Table 1:** Causes for secondary FSGS.

Group	Examples
Infections	HIV Parvovirus B19 CMV

Drugs	Heroine Bisphosphonate Calcineurin inhibitors and mTor inhibitors Interferon

Prenatal risk factors	Very low birth weight Intrauterine growth retardation

Life style	Obesity Anabolic steroids

Reduction in nephron mass	Resection Trauma

Tubulointerstitial injury	Reflux nephropathy Dent's disease Lowe syndrome

Systemic diseases	Plasma cell dyscrasias Weber-Christian Disease

Genetic mutations	Nephrin Podocin CD2AP WT1 Laminin *β*2 Alpha-actinin 4
